# B4GALNT1 induces angiogenesis, anchorage independence growth and motility, and promotes tumorigenesis in melanoma by induction of ganglioside GM2/GD2

**DOI:** 10.1038/s41598-019-57130-2

**Published:** 2020-01-27

**Authors:** Hideki Yoshida, Lisa Koodie, Kari Jacobsen, Ken Hanzawa, Yasuhide Miyamoto, Masato Yamamoto

**Affiliations:** 1 Department of Surgery, University of Minnesota, Minneapolis, Minnesota USA; 2 Department of Molecular Biology, Osaka International Cancer Institute, Osaka, Japan; 3 Masonic Cancer Center, University of Minnesota, Minneapolis, Minnesota USA; 4 Stem Cell Institute, University of Minnesota, Minneapolis, Minnesota USA

**Keywords:** Melanoma, Glycobiology, Cell migration, Cancer genetics, Tumour angiogenesis

## Abstract

*β-1,4-N-Acetyl-Galactosaminyltransferase 1 (B4GALNT1)* encodes the key enzyme B4GALNT1 to generate gangliosides GM2/GD2. GM2/GD2 gangliosides are surface glycolipids mainly found on brain neurons as well as peripheral nerves and skin melanocytes and are reported to exacerbate the malignant potential of melanomas. In order to elucidate the mechanism, we performed functional analyses of B4GALNT1-overexpressing cells. We analyzed ganglioside pattern on four melanoma and two neuroblastoma cell lines by high performance liquid chromatography (HPLC). We overexpressed B4GALNT1 in GM2/GD2-negative human melanoma cell line (SH4) and confirmed production of GM2/GD2 by HPLC. They showed higher anchorage independence growth (AIG) in colony formation assay, and exhibited augmented motility. *In vitro*, cell proliferation was not affected by GM2/GD2 expression. *In vivo*, GM2/GD2-positive SH4 clones showed significantly higher tumorigenesis in NOD/Scid/IL2Rγ-null mice, and immunostaining of mouse CD31 revealed that GM2/GD2 induced remarkable angiogenesis. No differences were seen in melanoma stem cell and Epithelial-Mesenchymal Transition markers between GM2/GD2-positive and -negative SH4 cells. We therefore concluded that B4GALNT1, and consequently GM2/GD2, enhanced tumorigenesis via induction of angiogenesis, AIG, and cell motility. RNA-Seq suggested periostin as a potential key factor for angiogenesis and AIG. These findings may lead to development of novel therapy for refractory melanoma.

## Introduction

Malignant melanoma is the most common and lethal skin cancer^1,2^. It is a cancer with one of the biggest rise in incidence^3,4^, and the overall 5-year survival rate is less than 10% for patients with stage IV disease^5,6^. There have been major advances in the treatment of advanced melanoma including Ipilimumab, an antibody to cytotoxic T-lymphocyte-associated-antigen-4 (CTLA-4), and BRAF inhibitor^7–9^. However, the anti-CTLA-4 antibody shows benefit in less than 50% of patients^10^. While BRAF inhibitors increased survival compared to other chemotherapies, its indication is limited to about half of patients with BRAF V600 mutations, and almost all patients develop resistance to these inhibitors^11^. While the combination of Nivolumab (monoclonal antibody against programmed death 1, PD-1) and Ipilumumab has demonstrated an impressive 2-year overall survival rate of 63.8% in stage III-IV patients^12^, further improvement of therapy is still needed for the treatment of advanced melanoma patients.

**Figure 1:**
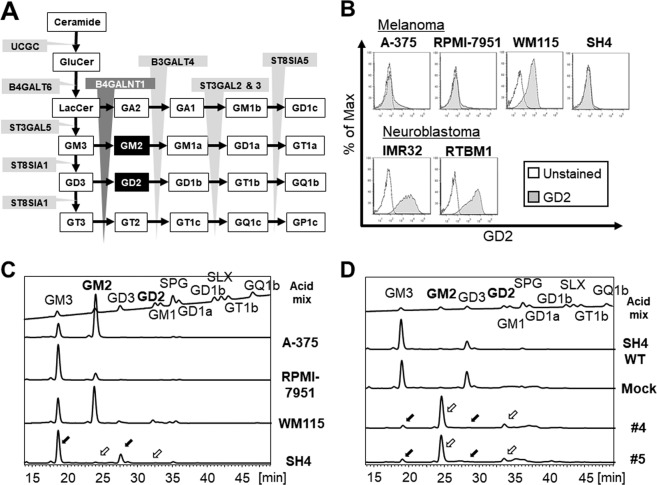
Schemes of ganglioside synthesis and analyses of gangliosides in the cells. (**A**) Glycosylation sequences for biosynthesis of GM2/GD2. B4GALNT1 (*β-1,4-N-Acetyl-Galactosaminyltransferase 1*) is the critical enzyme for the GM2/GD2 synthesis. (**B**) Flow cytometry Analysis of GD2 on the cultured cells; four human melanoma cell lines (A375, RPMI-7951, WM-115 and SH4), and two human neuroblastoma cell lines (IMR32 and RTBM1). (**C**) Detail analysis of acidic gangliosides on the four melanoma cell lines. The surface expression of gangliosides was analyzed by HPLC. Y-axis indicated intensity of fluorescence. (**D**) HPLC-based analysis of acidic gangliosides on SH4 cell line before and after B4GALNT1 overexpression. Mock is SH4 with pcDNA3.1(+) expression vector alone. #4 and #5 single cells are isolated SH4 clones with B4GALNT1 overexpression. Black arrows; GM3/GD3, white arrows; GM2/GD2.

*β-1,4-N-Acetyl-Galactosaminyltransferase 1 (B4GALNT1)* encodes B4GALNT1 (GM2/GD2 synthase), and it works as the key enzyme which transfers a N-acetylgalactosamine (GalNAc) to GM3/GD3, yielding gangliosides GM2/GD2 as part of their stepwise synthesis (Fig. 1A). Gangliosides, including GM2 or GD2, belong to the family of glycosphingolipids (GSL) and contain one or more sialic acids, N-acetyl derivatives of neuraminic acid, in their hydrophilic oligosaccharide chain.^13^ Gangliosides are sialic acid-containing glycosphingolipids that are most abundant in the nervous system, especially brain neurons^14^. They also exist in peripheral nerves and skin melanocytes^15,16^. These molecules are reported to have important biological functions, such as intercellular communication, cell cycling, cell growth, adhesion, differentiation, and cell motility^17–19^. Gangliosides are not only detected at high levels in tumors of neuroectodermal cell origin but also related to the biological and clinical behavior of many kinds of tumors^20^. Recently, some analysis revealed that patients with higher expression of B4GALNT1 and GM2/GD2 correlated with poorer prognosis in renal cell carcinoma (TCGA data set; Human Protein Atlas), neuroblastoma^21^, and melanoma^22^. Thus, B4GALNT1 gene is considered to be key tumor-associated antigens^23–27^, indicating that their expression is a meaningful marker for metastatic condition and are potential therapeutic targets for melanoma.

Our findings indicate the involvement of B4GALNT1 and GM2/GD2 in tumor establishment and progression as well as a potential direction of therapeutic approach *via* controlling B4GALNT1, and consequently GM2/GD2 expression in cancers such as melanoma.

## Results

### GM2/GD2 expression status in melanoma and neuroblastoma cell lines

To assess the GM2/GD2 expression level, four melanoma (A-375, RPMI-7951, WM115 and SH4) and two neuroblastoma cell lines (IMR32 and RTBM1) were measured by flow cytometry. One melanoma (WM115) and both of two neuroblastoma cell lines expressed high level of GM2/GD2 (Fig. 1B).

Because gangliosides including GM2/GD2 require stepwise synthesis reactions (Fig. 1A), a model for induced expression of GM2/GD2 on cell surface via overexpression of B4GALNT1 needs the following conditions; 1) both GM3 and GD3 are positive, and 2) both GM2 and GD2 are negative. To evaluate these conditions accurately in the six cell lines, HPLC-based high-specificity analysis of gangliosides was performed (Fig. 1C). Being that SH4 melanoma cell line showed high expression of both GD3 and GM3 (black arrows) and no expression of GD2 and GM2 (white arrows), SH4 fulfilled the aforementioned conditions and was used in the following study. Other results of neuroblastoma cells were shown in Fig. S1.

### Generation of GM2/GD2-positive SH4 melanoma clones

The SH4 cells were transfected with expression vectors with or without *B4GALNT1* gene cassette, to establish GM2/GD2-positive and -negative SH4 clones. Two GM2/GD2-high clones were selected by single cell isolation (#4 and #5, Fig. S2A). These two clones showed significant expression of GD2, whereas Mock (pcDNA3.1(+) alone) and two clones showed no GD2 expression. The expressions of *B4GALNT1* in mRNA level were in correspondence with those by flow cytometry (Fig. S2B). Additionally, HPLC revealed that the clones #4 and #5 expressed GM2/GD2 at high level (Fig. 1D). The reason that GD2 level in the transfected clones is very low compared to the GD3 level in the parental cells was interpreted that B4GALNT1 and ST8Sia1 competes GM3 as a substrate. It is known that GD2 is not synthesized from GM2^28^.

### Induction of morphological change, anchorage independence growth, and cell motility

**Figure 2:**
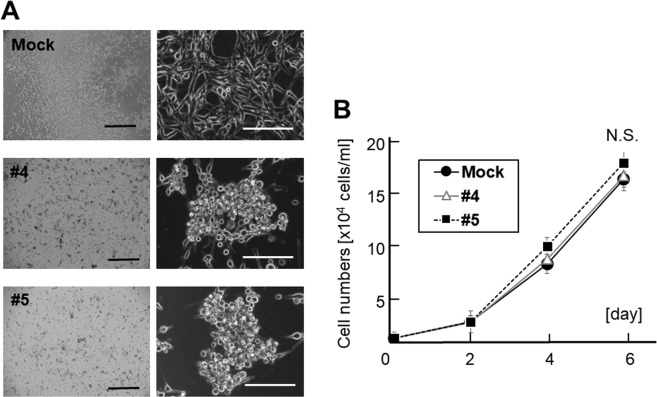
Effect of B4GALNT1 overexpression on cell morphology and growth. (**A**) Morphological changes in SH4 after B4GALNT1 overexpression. Scale bar, 1,000 μm (left) and, 200 μm (right). (**B**) Cell growth of SH4 with or without GM2/GD2-expression 6 days later. Results represent the means $\mp$ s.d. from three independent experiments.

**Figure 3:**
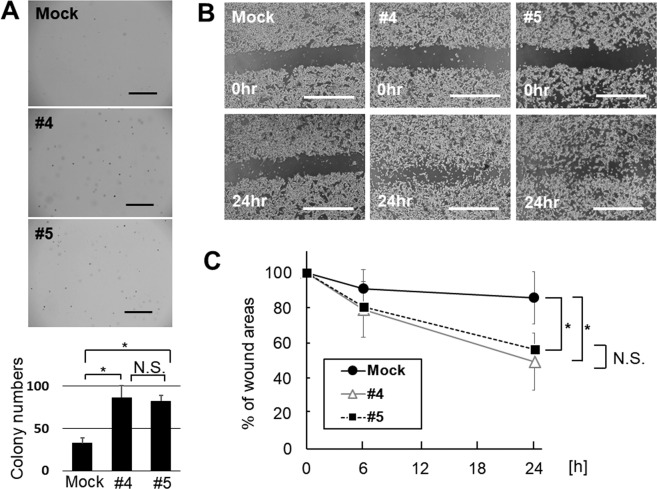
Effect of B4GALNT1 overexpression to cell behavior. (**A**) Photographs of colonies of SH4 cells with and without B4GALNT1 overexpression 14 days later. Scale bar, 1,000 μm. Results represent the means ∓ s.d. of three independent experiments. (**B**) Light microscopic images of SH4 cells that were scratched and compared the wound width 6 and 24 h later. Scale bar, 1,000 μm. (**C**) Average wound widths, expressed as a percent of the original width, obtained from 30 measurements in each photo. Results represent the means ∓ s.d. of three independent experiments. *P < 0.01 compared with Mock. N.S.; Not Significant.

The SH4 clones overexpressing GM2/GD2, #4 and #5, exhibited a distinct morphological appearance compared to SH4 Wild type (WT) or the mock transduced cells. The cells were round and formed aggregation. More than half of them were detached from the bottom of flask, but still capable of survival and proliferation after detachment (Fig. 2A). No significant difference was seen between the proliferation of GM2/GD2-positive SH4 clones and control (Fig. 2B). A soft agar colony formation assay demonstrated that GM2/GD2-positive SH4 clones formed larger and greater number of colonies than GM2/GD2-negative cells (#4; 86.6 ± 13.9, #5; 82.5 ± 6.5, Mock; 32.7 ± 6.6, #4 vs Mock; p < 0.0001, #5 vs Mock; p < 0.0001, Fig. 3A). There was no significant difference between the two GM2/GD2-positive SH4 clones (#4 vs #5; P = 0.15). In addition, faster wound closure was observed in the GM2/GD2-positive SH4 clones than the control cells (#4: 49.7 ± 16.4 vs #5: 56.5 ± 25.3 vs Mock: 85.9 ± 14.8, #4 vs Mock: p < 0.0001, #5 vs Mock: p < 0.0001, #4 vs #5: p = 0.98, Fig. 3B,C), indicating enhanced motility.

### Enhancement of tumor incidence and growth speed *in vivo*

**Figure 4:**
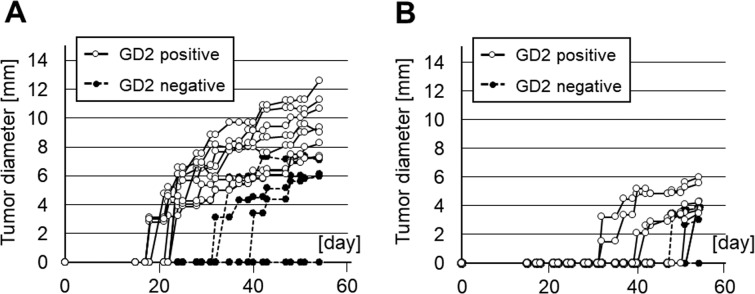
Effects of B4GALNT1-overexpressing on the proliferation of SH4 cells in xenograft models. (**A**) 2 × 10^6^, (**B**) 2 × 10^5^ SH4 cell were inoculated into lower flank of NSG mice subcutaneously.

To assess the *in vivo* effect of GM2/GD2 induced by B4GALNT1 overexpression, the two GM2/GD2-positive SH4 clones and Mock were inoculated in NOD/Scid/IL2Rγ-null (NSG) mice to assess tumor initiation and growth. After transplanting 2 × 10^6^ GM2/GD2-positive and -negative SH4 cells, all mice receiving #4 and #5 cells developed tumors, whereas only three out of six mice injected with Mock cells developed tumors (P = 0.038, Fig. 4A, Table 1). In the NSG mice transplanted with lower number (2 × 10^5^) of GM2/GD2-positive or -negative SH4 cells, seven out of eight mice injected with #4 and #5 cells developed tumors, whereas two out of six mice injected with Mock cells developed tumors (P = 0.038, Fig. 4B, Table 1). Moreover, in the NSG mice transplanted with 2 × 10^6^ GM2/GD2-positive or -negative SH4 cells, tumors derived from GM2/GD2-positive cells grew to be approximately three times larger than the GM2/GD2-negative Mock at day 54 (9.4 ± 1.9 vs 3.2 ± 3.5 mm, P = 0.003, Fig. 4A). In the NSG mice transplanted with 2 × 10^5^ GM2/GD2-positive or -negative SH4 cells, tumors derived from GM2/GD2-positive cells grew to be over three times as large as those from GM2/GD2- negative Mock in 54 days (4.0 ± 1.8 vs 1.1 ± 1.8 mm, P = 0.026, Fig. 4B). Every tumor was solid, firm, and partially fibrotic.

**Table 1:** Tumor incidence *in vivo*.

		GM2/GD2		p-value
		positive	negative	
cell	2 × 10^6^	8/8	3/6	P < 0.05
number	2 × 10^5^	7/8	2/6	P < 0.05

### No evident difference in major cancer stem cell markers

To evaluate the possibility that B4GALNT1 overexpression might enhance tumor incidence via induction of stemness, several melanoma stem cell markers were analyzed in GM2/GD2- positive and -negative cells by flow cytometry. Previous reports indicated that CD133^29–31^, CD166^29^, CD271^32^, Nestin^30^,and ABCB5^33^ are potential melanoma stem cell markers. Aldehyde dehydrogenase (ALDH) activity has also been reported as a potential marker of melanoma stem cell^34^. However, B4GALNT1 overexpression did not induce any meaningful change in any of these markers (Fig. S3A–F), there was no significant difference in CD133, CD166, and ABCB5 between GM2/GD2-positive and -negative cells. There was a small decrease in the expression of CD271 and Nestin in the #5 GM2/GD2-positive cell line, but this alone is unlikely to be the cause of enhanced tumor growth. That indicated that B4GALNT1 was unlikely to affect the stemness in the SH4 melanoma cell line.

### Promotion of angiogenesis *in vivo*

**Figure 5:**
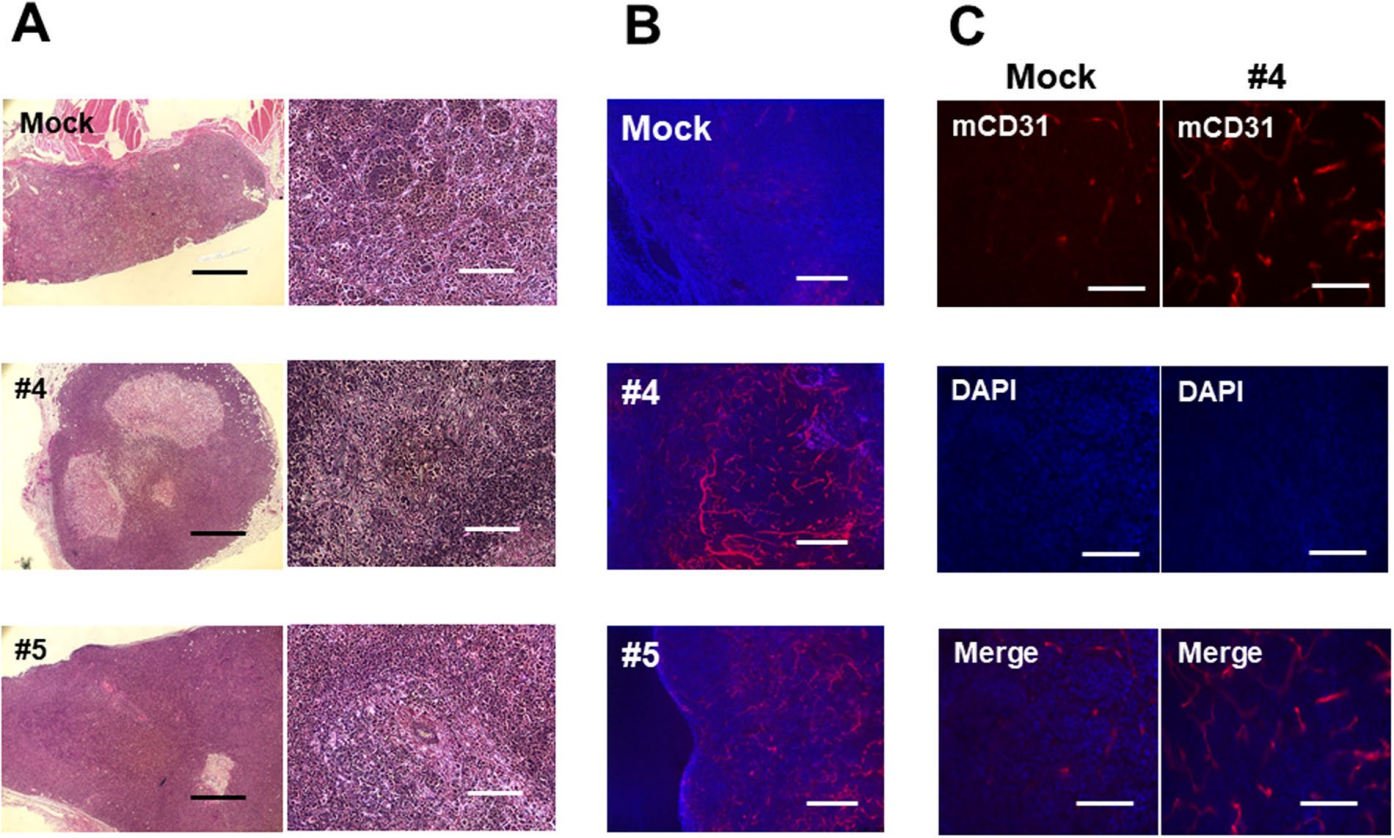
Histological analyses of the tumors. (**A**) Images of tissue sections of SH4 (H&E). (**B**,**C**) Immunostaining with murine CD31. Scale bar, 1,000 μm (**A**; left), 200 μm (**A**; right), 400 μm (**B**), and 100 μm (**C**), respectively.

We hypothesized that B4GALNT1 may enhance tumor induction and growth by increasing tumor vascularization. Tumors of similar size from each group were stained by hematoxylin and eosin (H&E) and immunofluorescence for murine-CD31. In H&E staining, the tumors induced by GM2/GD2-positive or -negative SH4 cells did not differ in any characteristics examined: cell shape, number of giant cells, nuclear-to-cytoplasm volume ratio reversal, and hyperchromatism (Fig. 5A). The major blood vessel in the GM2/GD2-positive tumors were more prominent than the one supplying the Mock tumor. Although some tumors showed evidence of local invasion, none of the tumor-bearing mice developed metastasis.

Immunofluorescence staining in tumors derived from GM2/GD2-positive clones by anti-mouse CD31 Ab exhibited many well-structured vessels, while GM2/GD2-negative Mock tumors had much less (Fig. 5B,C). Immunofluorescence performed using selective anti-human-CD31 antibody (non-reactive to mouse CD31) as a negative control did not show any staining of blood vessels (data not shown).

### RNA-Seq revealed potential key molecule downstream of B4GALNT1

To identify the difference of the transcriptional profile between GM2/GD2-positive and -negative cells, we compared them by RNA-Seq and analyzed using edgeR. There were a total of 26,484 genes detected in the individual libraries (Table S1), and 472 genes showed over +/− two-fold change between the two groups (Table S2). The heat map was shown in Fig. S4A. Among the 472 genes, 117 genes were up-regulated and 351 genes were down-regulated by B4GALNT1 overexpression. There was no significant difference between the two Mock clones (Fig. S4B), as well as between two GM2/GD2-positive samples (#4 and #5 clones; Fig. S4C). Genes exhibiting the Top 10 largest fold changes are listed in Table 2. The top up-regulated gene, *PTPRD*, is a member of the protein tyrosine phosphatase (PTP) family, and is known to be a signaling molecule that regulates a variety of cellular processes including cell growth, differentiation as a tumor suppressor^35^, which was often down-regulated in a variety of tumors. The second highest was *B4GALNT1*, suggesting that our overexpression of the gene in SH4 had succeeded. The third highest, *POSTN* (periostin), functions as a ligand for alpha-V/beta-3 and alpha-V/beta-5 integrins to support adhesion and migration^36–38^. In addition, it is known to increase angiogenesis. Furthermore, *CPVL* (Carboxypeptidase Vitellogenic Like), the top down-regulated gene was related to maturation of monocytes into macrophages^39^. Carboxypeptidases are a large class of proteases that act to cleave a single amino acid from the carboxy termini of proteins or peptides. The exact function of this protein, however, has not been determined. The second and third downregulated genes were melanoma-associated antigen 12 (*MAGEA12*^40^) and chondrosarcoma associated Gene 1 (*CSAG1*^41^) which are oncogenes, supposed to be overexpressed in tumors, but in this case they were down-regulated upon *B4GALNT1* overexpression.

**Table 2:** Top 10 of the greatest genes expression changes in SH4 with vs without GM2/GD2.

Symbol	Gene name	Expr Fold change	p-value
**Up-regulation**			
*PTPRD*	Protein tyrosine phosphatase receptor type D	735.0563	0.005722
*B4GALNT1*	Beta-1,4-N-acetyl- galactosaminyltransferase 1	359.3801	1.4E-150
*POSTN*	Periostin	286.3453	4.95E-26
*SERPINB2*	Serpin family B member 2	240.9988	7.94E-06
*HLA-DRB1*	human leukocyte antigen, class II, DR beta 1	228.4819	1.07E-05
*IL13RA2*	Interleukin 13 receptor alpha 2	191.2431	0.000616
*CSMD1*	CUB and sushi multiple domains 1	188.313	7.75E-05
*MYBPC1*	Myosin binding protein C type 1	182.0119	0.000805
*ADGRL2*	Adhesion G protein- coupled receptor L2	158.3556	4.73E-92
*TNFRSF14*	TNF receptor superfamily member 14	142.9792	0.011144
**Down-regulation**			
*CPVL*	Carboxypeptidase vitellogenic like	−1370.3	3.27E-37
*MAGEA12*	Melanoma-associated antigen 12	−1143.23	9.84E-16
*CSAG1*	Chondrosarcoma associated gene 1	−676.619	8.41E-12
*GIPC3*	PDZ domain containing family Member 3	−535.495	1.85E-20
*CPM*	Carboxypeptidase M	−484.607	3.42E-15
*ZXDA*	Zinc finger, X-linked, duplicated A	−457.61	9.68E-09
*FAM83H*	Family with sequence similarity 83	−412.334	1.45E-08
*PRAME*	Preferentially expressed antigen in melanoma	−411.078	0.000851
*GABRQ*	Gamma-aminobutyric acid type A receptor theta subunit	−402.747	1.57E-21
*LDB2*	LIM domain binding 2	−371.463	0.000011

To further elucidate the role of the 472 genes, we categorized them by function related to cancer malignancy using Ingenuity Pathway Analysis (IPA). This included several genes that functioned as melanoma incidence, proliferation, mobility and colony formation. Some genes were up- or down-regulated as the past reports and others were not (Table 3; “*” means that the fold changes went the opposite direction compared with previous findings). Some of them changed in the opposite direction compared to past reports in melanoma carcinogenesis. These genes may not function as downstream of B4GALNT1, and may have shown a change opposite of other literature due to negative feedback. Pathway analysis was attempted, but no known pathway was found that could fully explain our expression profile suggesting that the revelation of the pathway downstream of glycolipids is not complete.

**Table 3:** Gene expression characterestics.

Symbol	Expr Fold change	findings	Symbol	Expr Fold change	findings
Up-regulatation			Down-regulated		
**Invasion of tumor**					
*POSTN*	286.345	Increases	*EDN3*	−248.929	Decreases
*NRP1*	117.774	Increases	VCAN*	−243.495	Increases
IGFBP5*	26.687	Decreases	*IL24*	−15.763	Decreases
*MMP1*	20.329	Increases	SERPINE1*	−9.371	Increases
TGFBR2*	6.627	Decreases	*SFRP1*	−6.729	Decreases
*ITGA1*	4.868	Increases	MMP2*	−6.046	Increases
*TBX2*	2.561	Increases	GPC1*	−4.435	Increases
HMGB3*	2.021	Decreases	CEACAM1*	−4.131	Increases
*SPHK1*	2.012	Increases	EGF*	−3.683	Increases
CTGF*	−2.077	Decreases	CSF1*	−2.879	Increases
**Migration of melanoma cell**					
NRP1*	117.774	Decreases	*VCAN*	−243.495	Affects
*SDC2*	11.904	Increases	L1CAM*	−20.041	Increases
*TNC*	2.217	Increases	SERPINA5*	−10.969	Increases
*SPHK1*	2.012	Increases	SERPINE1*	−9.371	Increases
			MMP2*	−6.046	Increases
			EGF*	−3.683	Increases
			FN1*	−2.15	Increases
**Cell proliferation of tumor cell lines**					
*POSTN*	286.345	Increases	PRAME*	−411.078	Increases
SERPINB2*	240.999	Decreases	VCAN*	−243.495	Increases
*IL13RA2*	191.243	Affects	*EMILIN2*	−124.892	Decreases
*NRP1*	117.774	Affects	TRPM2*	−94.449	Increases
*HTN1*	60.939	Increases	SYNM*	−26.275	Increases
MAF*	50.99	Decreases	PDGFB*	−22.149	Increases
*POU4F1*	50.781	Increases	L1CAM*	−20.041	Increases
*VIP*	39.551	Increases	CARD10*	−16.191	Increases
*CDK14*	30.195	Increases	*IL24*	−15.763	Decreases
*IGFBP5*	26.687	Increases	S100A4*	−15.312	Increases
**Colony formation**					
*POSTN*	286.345	Affects	VCAN*	−243.495	Increases
HTN3*	98.45	Decreases	*NUPR1*	−31.659	Decreases
*VIP*	39.551	Increases	PDGFB*	−22.149	Increases
*TP63*	8.144	Increases	*IL24*	−15.763	Decreases
*CDCA7L*	4.871	Increases	S100A4*	−15.312	Increases
*ITGA1*	4.868	Increases	*SERPINE1*	−9.371	Decreases
*ALDH1A1*	4.81	Affects	TBX3*	−7.463	Increases
*HAS2*	4.155	Increases	DUSP5*	−6.896	Increases
*TNC*	2.217	Affects	SFRP1*	−6.729	Increases
LIMA1*	2.133	Decreases	PTGES*	−6.519	Increases

*means that the fold changes went the opposite direction compared with previous findings.

## Discussion

In this study, we analyzed a variety of changes in the SH4 melanoma cell line upon overexpression of GM2/GD2 by transfection of *B4GALNT1 gene*. One of remarkable findings *in vitro* is that GM2/GD2-positive SH4 cells showed significant difference of AIG compared to Mock (Fig. 3A), interestingly, Mahata *et al*. also revealed that GM2/GD2 is associated with AIG by knocking out GM2/GD2 synthase^42^. AIG is often reported as a critical factor for tumorigenesis or exacerbation of malignancy^42–44^. Although we initially expected contribution of Epithelial-Mesenchymal Transition (EMT), neither *CDH1* (E-cadhelin) nor *VIM* (vimentin), the major EMT markers, showed change at the mRNA level (data not shown). On the other hand, our RNA-Seq result showed that the expression of *POSTN* in B4GALNT1-overexpressing cells increased by almost 300 times than Mock cells. Periostin is generally known as a cancer suppresser^45^ and it also help to migration in neuronal cell development^46^. While periostin is involved in numerous biological processes, it sometimes contributes to tumorigenesis by promoting cancer cell survival, invasion, and metastasis actively^36–38^. It is also known that high expression of periostin protein and/or mRNA is detected in variety of solid tumors^38,47^. Kudo *et al*. showed that periostin overexpression promoted invasion in head and neck squamous cell carcinoma cells^48^ and to explore the genes that are coordinately expressed with periostin, they performed microarray analysis. Among the genes changed in their study, *SULF1* was upregulated clearly in our result as well (9.30-fold; Table S2). On top of that, Kotbuki *et al*. directly revealed that periostin increased cell proliferation and invasion in melanoma cell *in vitro* and *in vivo* using overexpression system^49^, and Fukuda *et al*. showed that periostin was a key factor in promoting melanoma cell metastasis using shRNA^50^. We therefore speculated that the findings support our conclusion that periostin and its downstream gene overexpression promoted migration induced by GM2/GD2. Furthermore, Bao *et al*. demonstrated that periostin activated the downstream Akt/PKB pathway *via* αvβ3 integrin, by in which they observed phosphorylation of Akt1/PKBα on Ser473 to promote cellular survival in colon cancer^51^. Their phosphorylation level, not the total amount, would therefore be contributing to the downstream effect of GM2/GD2. This may explain why our RNA-Seq result did not show a remarkable fold change in Akt/PKB pathway (Table S1).

In our observation, B4GALNT1 overexpression did not affect cell proliferation *in vitro*, while multiple genes related to tumor cell proliferation-promoting, such as *POSTN, IL13RA2, NRP1* were up-regulated, and *EMILIN2*, which is a proliferation-suppressing gene, was down-regulated notably in mRNA level (Fig. 2B, Table 3). The relationship between ganglioside and cell proliferation is still controversial; some research suggest promotion of proliferation by gangliosides^52–54^, while others show inhibition^55^. These discrepancies may be explained by the difference in the ratio of GD2 + 3 vs GM2 + 3, as well as GD2 vs GD3, which may contribute to differences in cell proliferation. The report by Shibuya *et al*. which induced GM2/GD2 like ours showed enhanced cell migration^55^, similar to what we observed, supporting that B4GALNT1 and consequently GM2/GD2 intensified migration of SH4 cell (Fig. 3B). Periostin is known to promote motility of several different kinds of cells^56–58^. While our IPA analysis did not include the category of melanoma cell migration, RNA-Seq indicated that *SDC2* and *VCAN* might have led to the motility (Table 3).

Another finding was that GM2/GD2 strongly induced angiogenesis. The effect of B4GALNT1 for tumor incidence *in vivo* was assessed by injecting GM2/GD2-positive SH4 cells into NSG mice. As shown in Fig. 4A,B, the tumors injected GM2/GD2-positive cells showed a higher tumor establishment rate. This result corresponds with the fact that *B4GALNT1* is a clinical marker for advanced melanoma^59^. Liu Y *et al*. revealed that gangliosides accelerate tumor angiogenesis in murine cells and demonstrated that GM2/GD2-negative cells formed much smaller tumors, using GM3 synthase and GM2 synthase double knockout low ganglioside tumor model^60^. We assessed murine-CD31 expression in the tumors derived from GM2/GD2-positive cell by immunofluorescence staining and observed that B4GALNT1-overexpressing clones induced many CD31 positive endothelial cells and well-developed vessels. In addition, the surface of the GM2/GD2-positive tumors were better vascularized than that of Mock by observation of the recovered tumor with eyes. Tumor progression requires endothelial cells to be activated for the formation of a vascular system. Lang Z *et al*. found that the enrichment of human umbilical vein endothelial cell (HUVEC) membranes with ganglioside results in amplified VEGF-induced signaling that is important for angiogenesis, and concluded that ganglioside enhances VEGF-induced endothelial cell proliferation^61^. Liu Y *et al*. reported that reduction of gangliosides depleted vascularization, while addition of wild type gangliosides restored angiogenesis of ganglioside-poor tumor^60^. To clarify how gangliosides induce blood vessels, we assessed the relations of GM2/GD2 and VEGF. Some other reports indicate that ganglioside enhances VEGF and induces endothelial cell proliferation^61,62^. In addition, Liu Y *et al*. also reported that periostin induces angiogenesis via Erk/VEGF pathway^63^. However, in our result of RNA-Seq and real-time RT-PCR, the expression of VEGF did not show significant correlation with GM2/GD2 level (Table S1, Fig. S5). There is a possibility that interaction between periostin and integrins directly promoted angiogenesis^64^ or GM2/GD2 lowered the threshold for cytokine stimulation^60,65^.

While GM2 and GD2 were reported to be increased greatly in cancer stem cells in breast cancer^66,67^, our data ruled out the possibility that GM2/GD2 enhanced tumor incidence via induction of cell stemness. We assessed some melanoma stem cell markers, such as CD133, CD166, CD271, Nestin, ABCB5, and ALDH activity by flow cytometry, and there was no evidence indicative of GM2/GD2 involvement in stemness (Fig. S3A–F).

In summary, our findings demonstrated that in the SH4 melanoma cell line, overexpression of B4GALNT1 as well as its main products GM2/GD2 promotes AIG and cell migration *in vitro* and enhances tumor incidence by inducing angiogenesis *in vivo*. To our best knowledge, this is the first time that RNA-Seq was performed to elucidate the influence of B4GALNT1. This result indicates that GM2/GD2 or B4GALNT1 is upstream regulator of periostin, and it might cause some change of characters related to tumorigenesis mentioned above in melanoma cell line. In this study, we have not only shown how GM2/GD2 exacerbates tumors’ malignant characters by using B4GALNT1 artificial expression system, but also reconfirmed RNA-Seq is useful tool to find novel potential target in cancer.

## Materials and Methods

All experiments were performed in accordance with relevant guidelines and regulations.

### Cell lines

Human melanoma cell lines (A375, RPMI-7951, SH4 and WM115) were purchased from American Type Culture Collection (Manassas, USA). Human neuroblastoma cell lines (IMR32 and RTBM1) were provided by Dr. Hajime Hosoi (Kyoto Prefectural University of Medicine, Japan). The melanoma cell lines were maintained in Dulbecco’s modified Eagle’s high-glucose medium (DMEM, Corning, USA) and neuroblastoma cell lines were maintained in Eagle’s minimal essential medium (EMEM, Corning) supplemented with 10% FBS, 100 U/ml penicillin and 10 mg/ml streptomycin (Corning). HUVEC was maintained in Endothelial Cell Growth Media (Sigma-Aldrich, USA). All cells were cultured at 37 °C in a humidified atmosphere of 5% CO_2_.

### Construction of a cDNA expression vector, gene transfection and selection

Human *B4GALNT1* cDNA was cloned from IMR32 with the primers listed in Table S3. The fragment was first inserted into Topo vector using Zero Blunt TOPO PCR Cloning Kit (Invitrogen, USA). After confirmation of sequence, the cDNA cut out by BamHI and NotI was inserted into the cDNA3.1(+) expression vector (Invitrogen). SH4 cells were plated in a 60-mm plastic plate (Corning) and then transfected with the plasmids by using Superfect (Qiagen, Germany). Stable transfectants were isolated in the presence of 600 μg/ml G418 (Roche, Germany).

### Flow cytometric analysis

Cells were trypsinized and washed twice with flow cytometry buffer (FCB, PBS supplemented with 1% FBS and 0.02% sodium azide (Sigma-Aldrich)). Cells were incubated with the anti-hGD2 mAbs (MAB2052, Millipore, USA) and anti-hABCB5 mAbs (MA5-17026, Thermo Fischer Scientific, USA (1:100, 100 μl/10^6^ cells) for 1 h and then washed in FCB. The cells were subsequently incubated with FITC-labeled anti-mouse goat IgG (sc-2010; 1:1,000, 1 ml/10^6^ cells, Santa Cruz Biotechnology, USA) for 40 min, and washed twice with FCB. Cells were incubated in PE-conjugated anti-hCD133/2 mAbs (#130-090-853, Miltenyi Biotec, Germany), PE-conjugated anti-hALCAM/CD166 mAbs (#105902, R&D systems, USA) and FITC-conjugated anti-hCD271/NGFR antibody (#345103, BioLegend, USA), (1:200, 1 ml/10^6^ cells) for 15 min and then washed with FCB. With PE-conjugated anti-hNestin mAbs (#196908, R&D systems), cells (1 × 10^6^ cells/200 μl) were fixed with 200 μl of cold 4% paraformaldehyde (Sigma-Aldrich) at room temperature for 30 min, and then washed in FCB twice. After incubation with 500 μl 0.1% Triton X-100 (Sigma-Aldrich) for 10 min at room temperature, cells were washed with FCB twice and stained with the antibody (1:200, 1 ml/10^6^ cells) on ice for 30 min in the dark, and subsequently washed with FCB. ALDH activity was determined by ALDEFLUOR Kit (Stemcell Technologies, Canada) according to the manufacturer’s instructions. All procedures were performed at 4 °C. The samples were immediately analyzed using FACS Canto II flow cytometer (BD Bioscience, USA). In each sample at least 1 × 10^4^ events were collected. The data was analyzed with FlowJo software (FlowJo, LLC, USA).

### High-performance liquid chromatography (HPLC)

HPLC was carried out as described previously^68–70^. Briefly, the acidic glycosphingolipids were extracted from each melanoma and neuroblastoma cell line (1 × 10^6^ cells) and digested with recombinant endoglycoceramidase II from Rhodococcus sp. (Takara Bio, Japan). The released oligosaccharides were labeled with 2-aminopyridine and separated using a HPLC system equipped with a fluorescence detector. Normal-phase HPLC was performed on a TSK gel Amide-80 column (Tosoh, Japan). The molecular size of each PA-oligosaccharide is given in glucose units (Gu) based on the elution times of PA-isomaltooligosaccharides. Reversed-phase HPLC was performed on a TSK gel ODS-80Ts column (Tosoh). The retention time of each PA-oligosaccharide is given in glucose units based on the elution times of PA-isomaltooligosaccharides. Thus, a given compound on these two columns provides a unique set of Gu (amide) and Gu (ODS) values, which correspond to coordinates of the 2-D map. PA-oligosaccharides were analyzed using LC⁄ ESI MS⁄ MS. Standard PA-oligosaccharides, PA-GM1 and PA-GD1a, were purchased from Takara Bio and PA-LST-a and PA-SPG were obtained from our previous study^71^.

### Real time RT–PCR

Total RNA was extracted from a tumor specimen with RNeasy mini kit (Qiagen) and complementary DNA (cDNA) was synthesized by the use of the SuperScript VILO cDNA Synthesis Kit (Thermo Fisher Scientific) according to the manufacturer’s instructions, respectively. The primers used in this experiment are listed in Table S3. Real-time RT-PCR was carried out using LightCycler 480 System (Roche) with SYBR Green (Applied Biosystems, CA) as previously described^72^. Thermal cycling conditions were: initial denaturation for 10 min at 95 °C, and 40 cycles of 15 sec at 95 °C, and 1 min at 60 °C. Data were analyzed with the Light Cycler software.

### Assay for cell proliferation

Cells were seeded at 2 × 10^4^ cells/well in 12-well plate. Every 48 h, cells were dissociated by 0.25% trypsin (Corning) and neutralized by the same volume of 10% DMEM. After that 100 μl 0.5% trypan blue (Sigma-Aldrich) and counted by Cellometer Auto T4 (Nexcelom Bioscience, USA) until days 6.

### Anchorage-independent soft agar colony formation assay

Cells were cultured in a two-layer soft agar system^73^. It consisted of a 1% agarose (RPI, USA) underlayer and a 0.7% agarose overlayer containing 1 × 10^4^ cells in 60-mm dishes (Corning). Colonies were allowed to form for 2 weeks with fresh media added every 3 days. Plates were stained with crystal violet and colonies more than 0.1 mm in diameter were counted.

### Wound-healing assay

Wound-healing assays were carried out as described previously^74,75^. Immediately after scratching (0 h), the plates were photographed and the distance between the edges of the wound area was measured. At 6 h and 24 h after scratching, the plates were photographed and the distance between the edges of the wound region was again measured.

### *In vivo* tumorigenesis

Tumors were induced in 5–6 week old female and male NSG mice (Jackson Laboratory, USA). Each mouse was injected subcutaneously with SH4 cell lines transformed with pcDNA3.1(+) empty vector or the one expressing *B4GALNT1* suspended in 0.1 ml of PBS at a single site (2 × 10^6^ cell; left, 2 × 10^5^ cell; right) to the lower flank. Tumor diameter was monitored every 2–3 days on onset of tumor formation. Mice were sacrificed when the largest tumor size reached 16 mm in diameter along with IACUC approved protocol. At the end point of the experiments, tumors were extracted. At least 4 mice were used in each group (Mock, #4 and #5). The animal experiments were performed in accordance with the institutionally approved animal experimental protocol.

### Histopathology and immunohistochemistry

Histological specimens were fixed in 10% formalin and routinely processed for embedding in paraffin. The sections were stained with H&E. Some portions of tumors were embedded in OCT and frozen at −20 °C for immunofluorescence analyses. The tumors were sectioned 30-μm-thick by CM 1800 Cryostat (Leica Biosystems, Germany). The sections were fixed by 50 μl of ice-cold acetone for 5 min. After washing the slides in 1 x PBS, they were incubated in blocking buffer (2% bovine serum albumin (BSA) serum in PBS) for 1 h. The sections were incubated with PE-conjugated anti-mouse CD31 antibody (#102507, BioLegend, 1:500 at 4 °C overnight). After washing the slides in 1 x PBS three times, the slides were incubated by 20 μl VECTASHIELD Antifade Mounting Medium with DAPI (H-1200, Vector Laboratories, USA) and coverslipped. The tissues were observed with automated upright microscope System DM5500 B (Leica Biosystems).

### RNA sequencing (whole transcriptome shotgun sequencing, WTSS) and analysis

RNAs derived from SH4 with (#4 and #5) or without (Mock; two samples) *B4GALNT1* over-expression were analyzed by RNA sequencing as described in ref. ^76–78^. Quality tested with Bioanalyzer 2100 (Agilent Technologies, USA). Sequencing was accomplished on the MiSeq 500 (Illumina, USA). 50 bp FastQ paired-end reads (n = 23.6 Million per sample) were trimmed using Trimmomatic (v 0.33). Quality control checks on raw sequence data were performed with FastQC. Read mapping was performed via Hisat2 (2.1.0) using the Human UCSC genome (hg38) as reference. Differentially expressed genes were identified using the edgeR (Bioconductor, www.bioconductor.org) feature in CLCGWB (Qiagen) using raw read counts. The generated list was filtered based on a minimum 2 x absolute fold change and false discovery rate (FDR) corrected p < 0.05. Pathway analysis was performed in IPA (Qiagen) using fold change and FDR corrected values.

### Statistical analysis

Statistical analysis was performed using the unpaired Student’s t-test. A P-value of less than 0.05 was considered statistically significant.

## Supplementary information

Supplementary Figure 1.

Supplementary Figure 2A.

Supplementary Figure 3A .

Supplementary Figure 3B .

Supplementary Figure 3C .

Supplementary Figure 3D .

Supplementary Figure 3E .

Supplementary Figure 3F .

Supplementary Figure 4A .

Supplementary Figure 4B-C .

Supplementary Figure 5 .

Supplementary  Figure Legends .

Supplementary Table 1 .

Supplementary Table 2 .

Supplementary Table 3 .

.
