# Publisher Correction: B4GALNT1 induces angiogenesis, anchorage independence growth and motility, and promotes tumorigenesis in melanoma by induction of ganglioside GM2/GD2

**DOI:** 10.1038/s41598-020-74291-7

**Published:** 2020-10-09

**Authors:** Hideki Yoshida, Lisa Koodie, Kari Jacobsen, Ken Hanzawa, Yasuhide Miyamoto, Masato Yamamoto

**Affiliations:** 1 Department of Surgery, University of Minnesota, Minneapolis, Minnesota USA; 2 Department of Molecular Biology, Osaka International Cancer Institute, Osaka, Japan; 3 Masonic Cancer Center, University of Minnesota, Minneapolis, Minnesota USA; 4 Stem Cell Institute, University of Minnesota, Minneapolis, Minnesota USA

Correction to: *Scientific Reports* 10.1038/s41598-019-57130-2, published online 27 January 2020

In the original version of this Article the supplementary file was inappropriately formatted, resulting in multiple mislabelled files. This has been corrected in the HTML version of the Article; the PDF version was correct at time of publication.

