# Supplementary figures and images for: B4GALNT1 induces angiogenesis, anchorage independence growth and motility, and promotes tumorigenesis in melanoma by induction of ganglioside GM2/GD2
Source: Sci Rep. 2020 Jan 27;10:1199. doi: 10.1038/s41598-019-57130-2 (PMC6985110; doi:10.1038/s41598-019-57130-2)

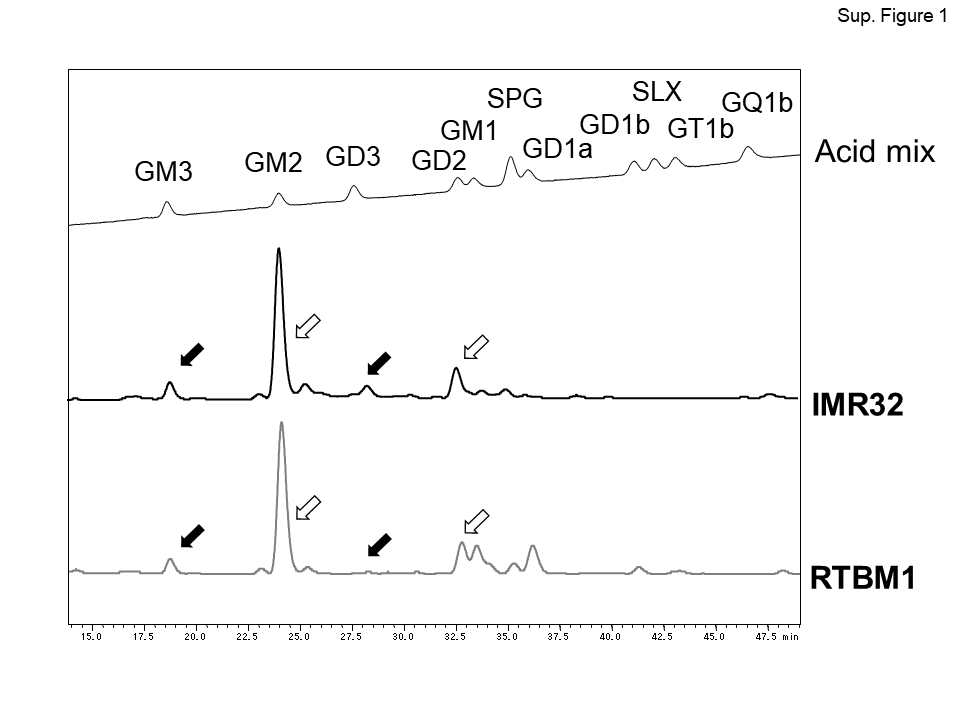

Supplement: Supplementary file 1 — Supplementary Figure 1. [file 41598_2019_57130_MOESM1_ESM.tif]

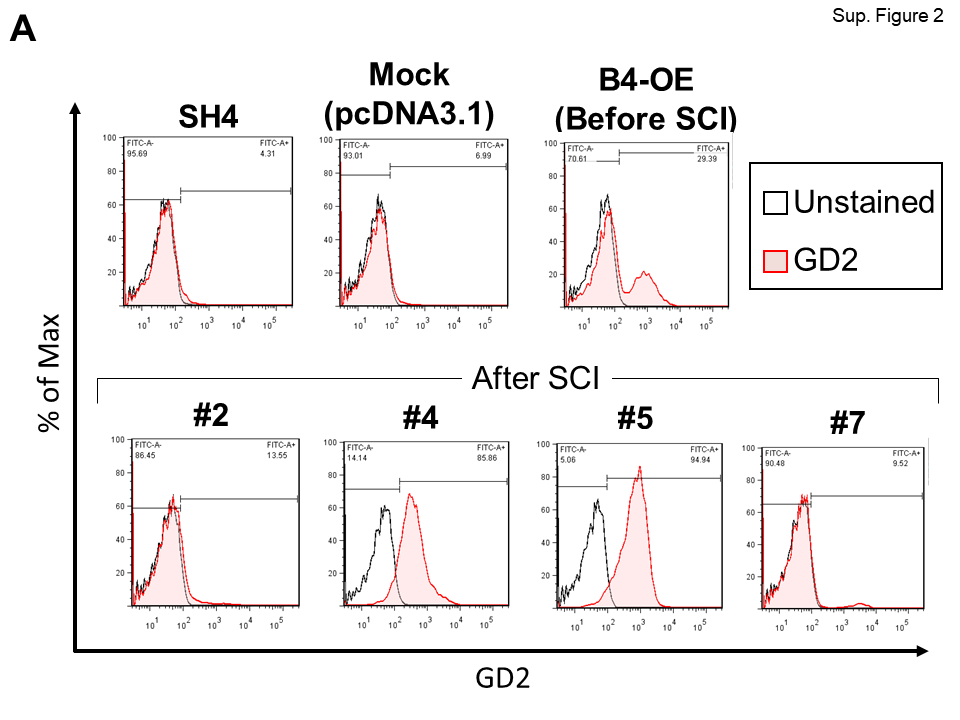

Supplement: Supplementary file 2 — Supplementary Figure 2A. [file 41598_2019_57130_MOESM2_ESM.tif]

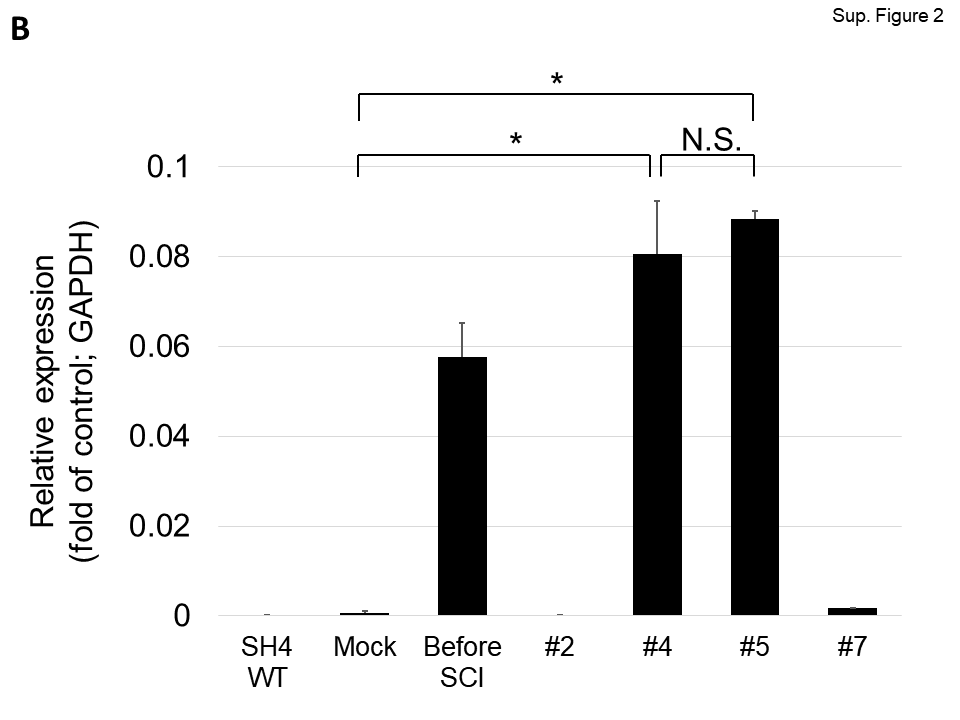

Supplement: Supplementary file 3 — Supplementary Figure 3A . [file 41598_2019_57130_MOESM3_ESM.tif]

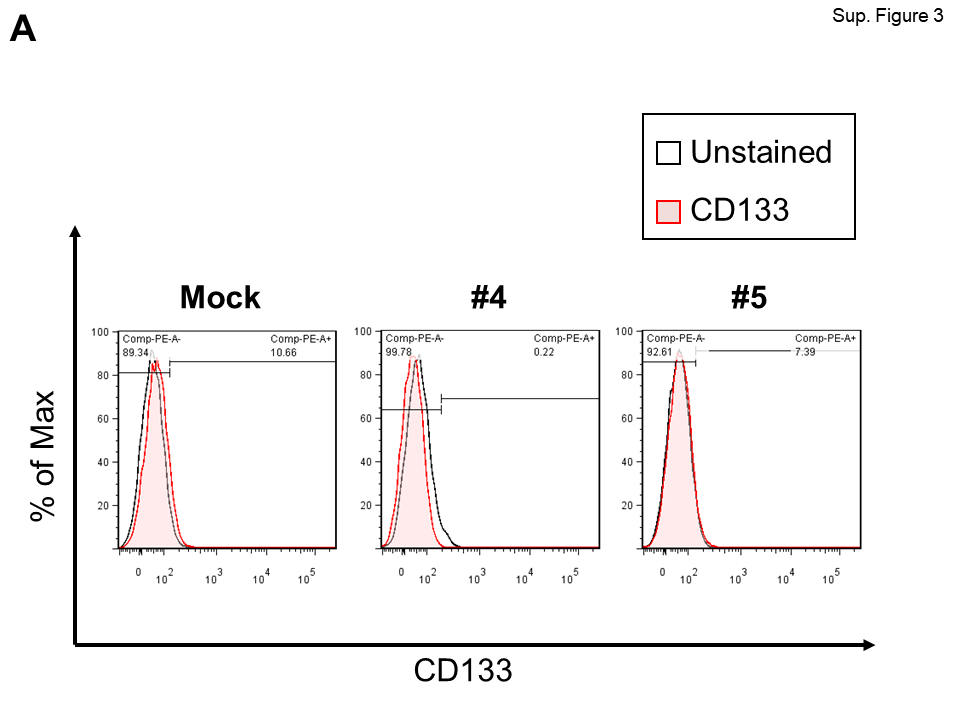

Supplement: Supplementary file 4 — Supplementary Figure 3B . [file 41598_2019_57130_MOESM4_ESM.tif]

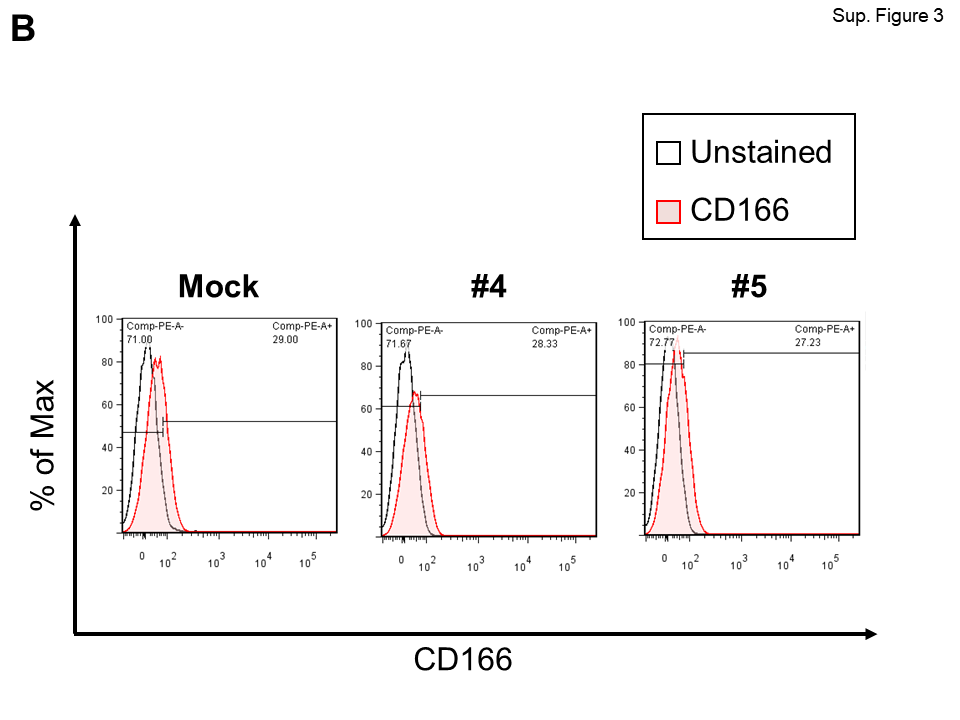

Supplement: Supplementary file 5 — Supplementary Figure 3C . [file 41598_2019_57130_MOESM5_ESM.tif]

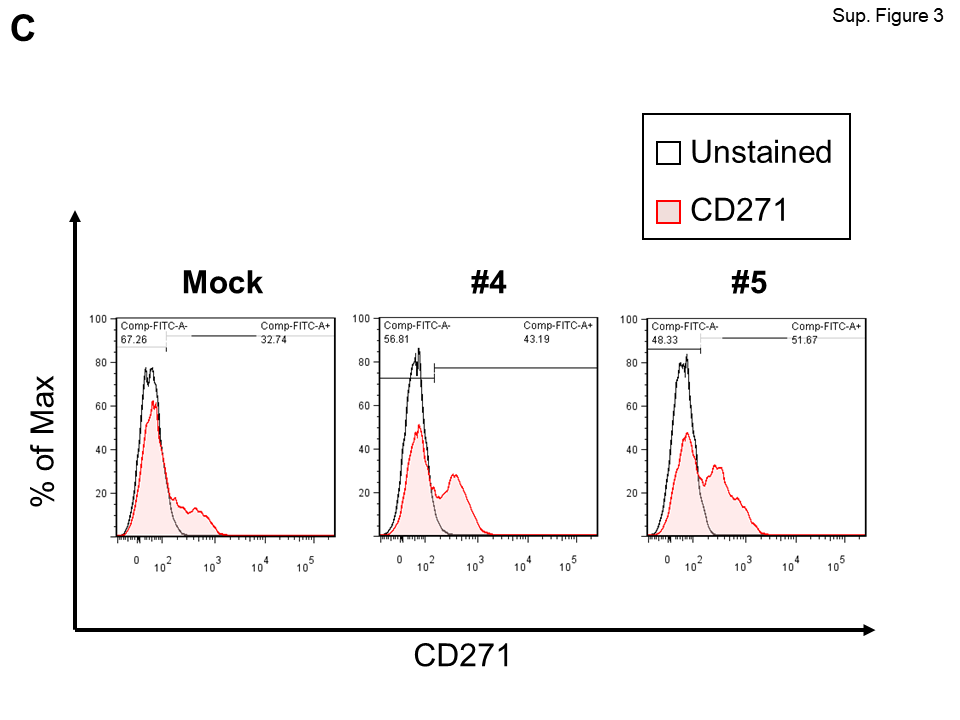

Supplement: Supplementary file 6 — Supplementary Figure 3D . [file 41598_2019_57130_MOESM6_ESM.tif]

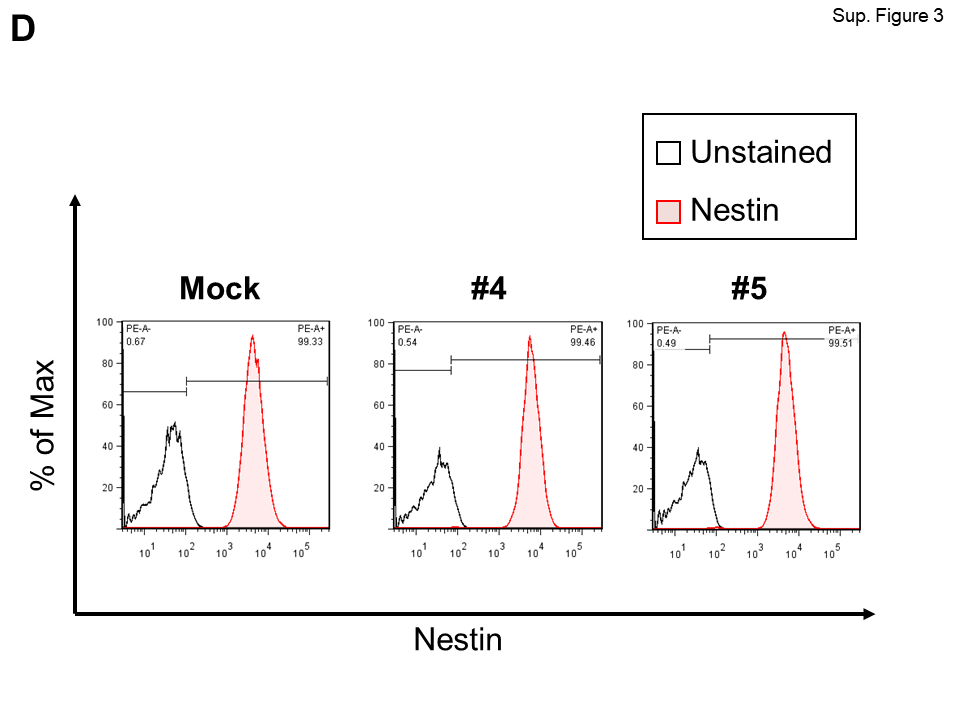

Supplement: Supplementary file 7 — Supplementary Figure 3E . [file 41598_2019_57130_MOESM7_ESM.tif]

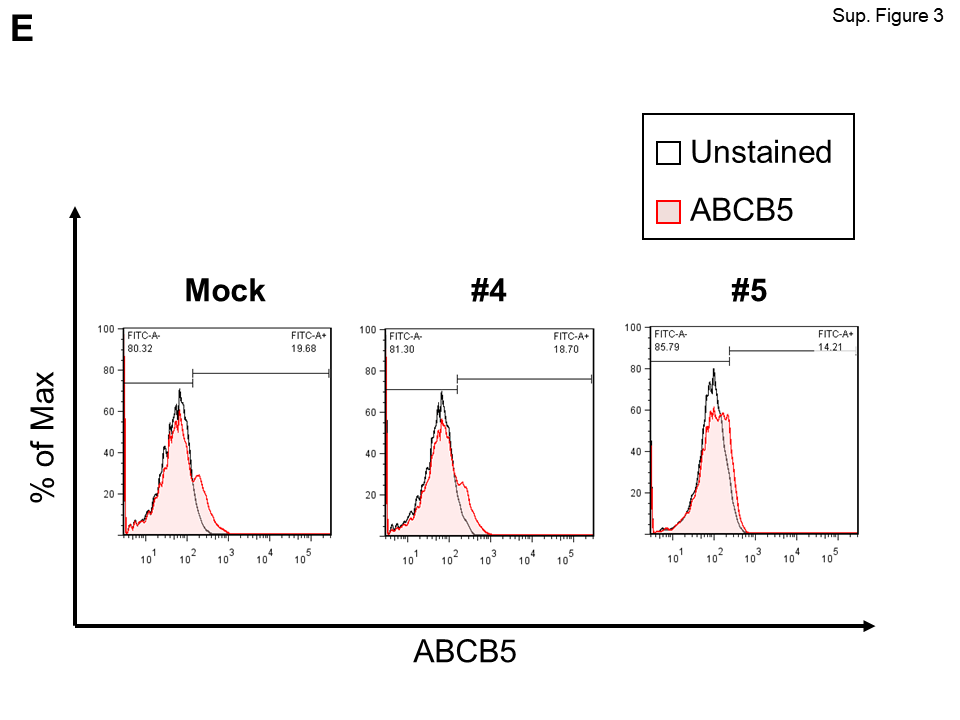

Supplement: Supplementary file 8 — Supplementary Figure 3F . [file 41598_2019_57130_MOESM8_ESM.tif]

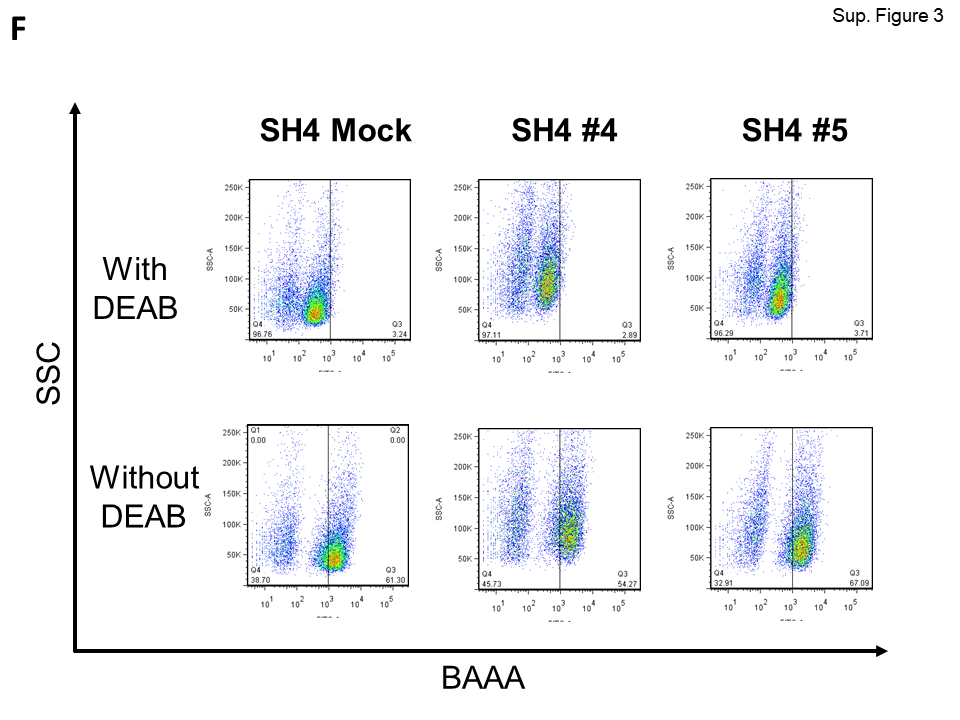

Supplement: Supplementary file 9 — Supplementary Figure 4A . [file 41598_2019_57130_MOESM9_ESM.tif]

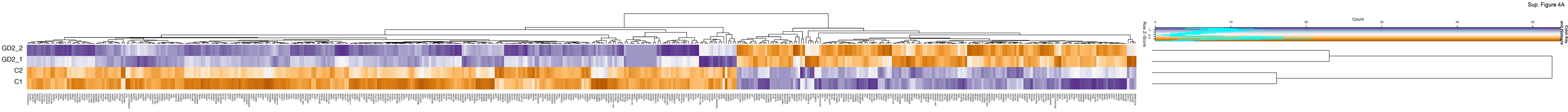

Supplement: Supplementary file 10 — Supplementary Figure 4B-C . [file 41598_2019_57130_MOESM10_ESM.tif]

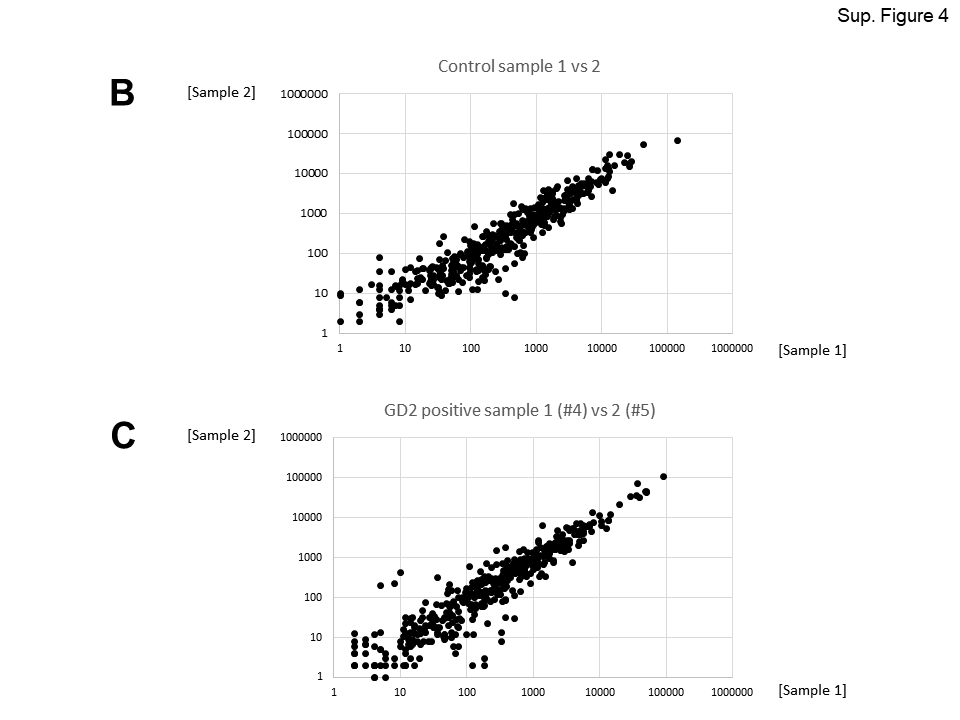

Supplement: Supplementary file 11 — Supplementary Figure 5 . [file 41598_2019_57130_MOESM11_ESM.tif]

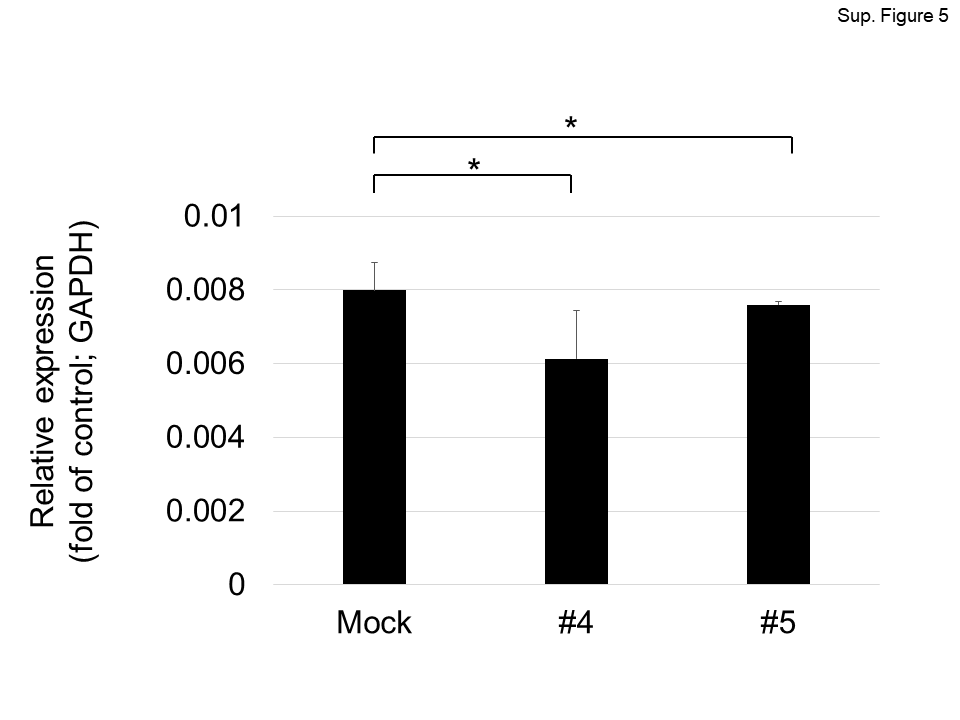

Supplement: Supplementary file 12 — Supplementary Figure Legends . [file 41598_2019_57130_MOESM12_ESM.tif]
