# Supplementary material for: B4GALNT1 induces angiogenesis, anchorage independence growth and motility, and promotes tumorigenesis in melanoma by induction of ganglioside GM2/GD2
Source: Sci Rep. 2020 Jan 27;10:1199. doi: 10.1038/s41598-019-57130-2 (PMC6985110; doi:10.1038/s41598-019-57130-2)
Supplement: Supplementary file 13 — Supplementary Table 1 . [file 41598_2019_57130_MOESM13_ESM.docx]

**Figure Legend for supplementary figure**

**Supplementary Figure 1. Detail analysis of acidic gangliosides on the two neuroblastoma cell lines.** The surface expression of gangliosides were analyzed by high performance liquid chromatography (HPLC). Y-axis indicated intensity of fluorescence. Black arrows means GM3/GD3, and white arrows means GM2/GD2, respectively.

**Supplementary Figure 2. Confirmation of GD2 expression in protein and mRNA level.**

(**A**) Expression of GD2 on the cell surface in SH4 cells before and after B4GALNT1 overexpression and single cell isolation. Mock; SH4 with pcDNA3.1(+) expression vector alone. B4-OE; B4GALNT1-overexpressing SH4 before single cell isolation. Four clones of SH4, #2, #4, #5 and #7 were single-cell-isolated after B4GALNT1 overexpression, only #4 and #5 showed high GD2 expression by flow cytometry.

(**B**) Relative expression of GD2 in mRNA level in SH4 cells before and after B4GALNT1 overexpression and single cell isolation. The expressions of GD2 in mRNA level were well correlated with those of FACS results. *; P<0.01 compared with Mock. N.S.; Not Significant.

**Supplementary Figure 3. The expression of melanoma stem cell markers in SH4 by flow cytometer.**

(**A**) CD133, (**B**) CD166, (C) CD271, (**D**) Nestin, (**E**) ABCB5, and (**F**) aldehyde dehydrogenase. SSC; side scatter, BAAA; BODIPY - aminoacetaldehyde, DEAB; diethylaminobenzaldehyde (ALDH selective inhibitor).

**Supplementary Figure 4. General result of RNA-Sequence.**

(**A**) A heat-map profile comparing B4GALNT1-overexpressing SH4 versus Mock by RNA-Seq. 472 genes were over ∓ two fold changed between these two groups.

(**B, C**) Dot blot comparing B4GALNT1-overexpressing SH4 versus Mock by RNA-Seq. (**B**) Control sample 1 vs 2; the same Mock clone, but RNA was extracted at the different passage point , (**C**) B4GALNT1-overexpressing samples 1(clone #4) vs 2(clone #5). There were no significant difference between two Mock samples, and between B4GALNT1-overexprssing two samples (#4 and #5 clones), respectively.
